# Long-Term Bending Behavior of Laminated Glass Plate with Temperature-Dependent Viscoelastic Interlayer

**DOI:** 10.3390/ma19132925

**Published:** 2026-07-07

**Authors:** Xia Zhu, Kangyu Ni, Changkuo Xu, Aiguo Zhao, Peng Wu

**Affiliations:** College of Civil Engineering, Nanjing Tech University, Nanjing 211816, China; zhuxia@njtech.edu.cn (X.Z.); nikangyu@njtech.edu.cn (K.N.); xuchangkuo@njtech.edu.cn (C.X.); shiang_37@163.com (A.Z.)

**Keywords:** laminated glass, viscoelastic interlayer, temperature-dependent behavior, state-space method, Laplace transform

## Abstract

This study presents an analytical model for the long-term bending behavior of simply supported laminated glass (LG) plates with temperature-dependent viscoelastic interlayers. The glass layers are described based on three-dimensional elasticity theory, and the governing stress and displacement equations are formulated using the state-space method. The polymer interlayer is characterized by the generalized Maxwell model and the Williams–Landel–Ferry equation, while its time-dependent response is described through the Boltzmann convolution principle. By combining double Fourier series expansions with the Laplace-transform technique, analytical solutions for the stresses and displacements of multilayer LG plates are derived. The comparison shows that Kirchhoff–Love plate theory gives results close to the present solution for relatively thin LG plates, whereas the discrepancy becomes increasingly pronounced as the plate thickness increases. The finite element results agree well with those obtained from the proposed model; however, for the representative benchmark case, the present solution is approximately 1.13 × 10^3^ times faster than the FE simulation, and its memory usage is only about 10.88% of that required by the FE model. Parametric studies further reveal the effects of temperature, interlayer thickness, interlayer material, number of glass layers, and aspect ratio on the stress redistribution and deflection development of LG plates.

## 1. Introduction

Laminated glass (LG) plates are important transparent structural components in modern civil engineering because they combine architectural transparency with enhanced safety and load-bearing capacity. They are widely used in facades, roofs, floors, and other transparent structural components. Compared with monolithic glass, LG plates can maintain post-breakage integrity because the polymer interlayer retains glass fragments and reduces the risk of collapse. However, the polymer interlayer exhibits significant viscoelastic behavior, and its mechanical properties depend strongly on both temperature and loading time. As a result, the shear-transfer capability of the interlayer may vary significantly during long-term service, which directly affects the bending stiffness, stress redistribution and deflection development of LG plates [1,2,3]. Therefore, an accurate evaluation of the long-term bending response of LG plates requires a refined model capable of simultaneously considering the time-dependent shear transfer of polymer interlayers and the influence of temperature.

Experimental investigations provide direct evidence for the mechanical behavior of LG plates and polymer interlayers. Vedrtnam et al. [4] studied the out-of-plane bending performance of ordinary LG panels under non-uniform temperature exposure, including pre-fracture, cracking and post-fracture stages. For two-layer LG plates under out-of-plane loading, Biolzi and Simoncelli [5] carried out experimental and numerical analyses to study the overall response of undamaged and damaged plates with different interlayers. Moving from plates to multi-layer members, Riddell-Smith et al. [6] tested 3-layer LG beams with different glass and interlayer types and analyzed their structural response and failure behavior. Elbelbisi et al. [7] showed through static tests and numerical simulations that larger LG panels had lower static resistance and larger deformation. Through tests on post-tensioned LG beams with hybrid reinforcement systems, Rocha et al. [8] found that this method improved their load-bearing capacity and post-cracking performance. Subsequently, Silvestru et al. [9] demonstrated through four-point bending tests that iron-based shape memory alloy tendons could enhance the initial and residual load-carrying capacity. Through out-of-plane bending tests and numerical calibration, Bedon et al. [10] showed that fractured glass fragments contributed to the residual stiffness of partially damaged two-layer LG elements. Santi and Royer-Carfagni [11] validated a fractional viscoelastic model for LG through four-point bending tests and relaxation tests at different temperatures. At the material level, Andreozzi et al. [12] characterized the thermo-viscoelastic properties of polyvinyl butyral (PVB) interlayers through dynamic torsion tests, and Pelayo et al. [13] fitted a Prony-based PVB model by means of static and dynamic tests combined with the Williams–Landel–Ferry (WLF) principle. Through shear tests at different temperatures, Biolzi et al. [14] investigated the viscoelastic response of PVB, SentryGlas and plasticized PVB in LG joints. In addition, Centelles et al. [15,16] characterized seven commercial interlayers through static relaxation tests and provided Prony parameters as well as storage and loss moduli; subsequently, long-term loading and recovery tests were conducted on LG slabs with different interlayers to study creep development, deflection recovery and residual deformation. These experimental studies indicate that interlayer material, temperature, loading duration and loading form can significantly affect the stiffness, deformation, damage and long-term response of LG, providing an important basis for subsequent theoretical modeling.

In addition to experimental investigations, analytical models are also widely adopted to analyze the mechanical behavior of LG plates, as they can reveal the underlying physical mechanisms, characterize load-transfer behavior, and enable efficient parametric studies beyond the limitations of experiments. Using a plane-beam model, Ivanov [17] analyzed the through-thickness stress and strain distributions of LG. With the interlayer represented by Prony-series viscoelastic models, Galuppi and Royer-Carfagni [18,19] analytically solved laminated beams and further discussed their response under time-dependent loading histories. Through a numerical approach, Jaśkowiec et al. [20] obtained displacement and stress fields of LG plates with different numbers of glass layers. For numerical implementation, Liang et al. [21] proposed an effective laminated shell element model for structural glass, in which the layered configuration and interlayer behavior are considered. From the viewpoint of refined structural theory, Naumenko and Eremeyev [22] developed a layer-wise shallow shell theory for LG and photovoltaic applications with a thin soft core. Baraldi [23] proposed a simple mixed finite element (FE) model for LG beams, where the interlayer shear stress is introduced as an independent variable to describe the shear interaction between glass layers. With respect to fracture simulation, Grozdanić et al. [24] developed a multiscale beam model for LG structures. In another formulation, Viviani et al. [25] introduced a fractional viscoelastic model for LG sandwich plates under blast actions. These studies have extended the analysis of LG from simplified beam models to refined shell, plate and numerical models, and have shown the importance of interlayer shear interaction, three-dimensional (3D) stress transfer and temperature-dependent viscoelasticity.

Although existing studies have provided experimental evidence and theoretical tools for LG analysis, several issues remain insufficiently addressed for LG plates. First, many theoretical models are still based on classical or simplified plate assumptions, in which transverse shear deformation and 3D stress components are simplified or neglected. Second, most existing analytical formulations are developed for two-layer or specific laminated configurations, and their extension to multilayer LG plates with arbitrary numbers of glass layers and interlayers is not straightforward.

To address these issues, this study develops an analytical model for the long-term bending response of LG plates with temperature-dependent viscoelastic interlayers. In the model, the 3D elasticity theory is employed to describe stresses and displacements, without introducing transverse shear deformation or equivalent-thickness assumptions. Second, the state-space method, transfer matrix method and Laplace transform are combined to derive the time-dependent stress and displacement fields of multilayer LG plates. Since the transfer matrix method can be assembled layer by layer, the proposed solution can be applied to multilayer LG plates with an arbitrary layer configuration. The proposed solution is then verified and used to investigate the effects of temperature, interlayer properties and geometric parameters.

## 2. Analytical Model

**Figure 1:**
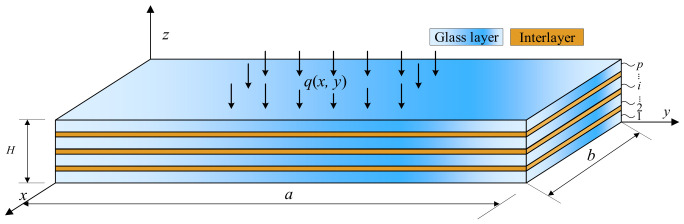
Schematic diagram of LG plate.

As illustrated in Figure 1, the LG plate considered in this study has a length *a*, a width *b*, and an overall thickness *H*. The plate consists of p glass layers, each with a thickness of *h_i_*, and *p* − 1 polymer interlayers of thickness Δ*h* placed between adjacent glass layers. In the following formulation, the superscript *i* denotes the *i*-th glass layer (*i* = 1, 2, …, *p*), whereas the superscript *k* denotes the *k*-th polymer interlayer (*k* = 1, 2, …, *p* − 1). The glass layers are isotropic linear elastic materials, whereas the polymer interlayers are temperature-dependent viscoelastic materials. The LG plate is simply supported along all four edges and is subjected to a transverse distributed load *q*(*x*, *y*) applied on the upper surface.

### 2.1. Assumptions and Limitations

The analytical model for the LG plate is developed under the following assumptions:

1. The LG plate undergoes linear deformation, and the glass layers remain intact and uncracked. The maximum principal tensile stress, strain, and transverse deflection are limited by the glass tensile strength, the strength-to-modulus ratio, and 1/100 of the shorter plate span, respectively. Therefore, cracked or fractured glass is beyond the scope of the present model.
2. Temperature is treated as an environmental variable affecting only the viscoelastic properties of the polymer interlayer, rather than as a thermal load. Thus, thermal stresses caused by temperature gradients, edge restraints, or manufacturing residual stresses are not considered. This assumption is suitable for approximately uniform service-temperature conditions with limited in-plane restraint, where the main temperature effect is the change in interlayer shear-transfer stiffness. For cases involving strong temperature gradients, restrained expansion, or significant production-induced residual stresses, thermal stresses should be included.

### 2.2. Equations for a Glass Layer

The mechanical behavior of glass layers in the LG plate is described using 3D elasticity theory [26], without invoking simplified plate assumptions associated with transverse shear deformation. Accordingly, the constitutive, geometric, and equilibrium equations of the *i*-th (*i* = 1, 2, …, *p*) glass layer can be written in tensor form as follows:

(1) $$\sigma_{j}^{i}=c_{jk}^{i}\gamma_{k}^{i},$$

(2) $$\gamma_{kj}^{i}=0.5(u_{k,j}^{i}+u_{j,k}^{i}),$$

(3) $$\sigma_{kj,j}^{i}=0,$$

where

$$[\sigma^{i}]=\left[\begin{array}{c} \sigma_{x}^{i}\\ \sigma_{y}^{i}\\ \sigma_{z}^{i}\\ \tau_{yz}^{i}\\ \tau_{xz}^{i}\\ \tau_{xy}^{i} \end{array}\right],\;[c^{i}]=\left[\begin{array}{cccccc} \lambda^{i}+2G^{i} & \lambda^{i} & \lambda^{i} & 0 & 0 & 0\\ & \lambda^{i}+2G^{i} & \lambda^{i} & 0 & 0 & 0\\ & & \lambda^{i}+2G^{i} & 0 & 0 & 0\\ & & & G^{i} & 0 & 0\\ & sym. & & & G^{i} & 0\\ & & & & & G^{i} \end{array}\right],\;[\gamma^{i}]=\left[\begin{array}{c} \varepsilon_{x}^{i}\\ \varepsilon_{y}^{i}\\ \varepsilon_{z}^{i}\\ \gamma_{yz}^{i}\\ \gamma_{xz}^{i}\\ \gamma_{xy}^{i} \end{array}\right],\;[u^{i}]=\left[\begin{array}{c} u_{x}^{i}\\ u_{y}^{i}\\ u_{z}^{i} \end{array}\right].$$

in which *λ^i^* and *G^i^* are the Lamé constants, which can be expressed by the elastic modulus *E^i^* and Poisson’s ratio *ν^i^*:

(4) $$\lambda^{i}=\frac{E^{i}\nu^{i}}{\left(1+\nu^{i})(1-2\nu^{i}\right)},\;G^{i}=\frac{E^{i}}{2(1+\nu^{i})}.$$

The transverse normal stress $\sigma_{z}^{i}$ is retained in the proposed 3D elasticity formulation because 3D elasticity theory naturally provides the complete stress, strain and displacement fields, rather than prescribing simplified stress assumptions in advance. In addition, $\sigma_{z}^{i}$ is required to satisfy the full 3D equilibrium equations and the surface traction boundary conditions. Its consideration also improves the completeness and generality of the analytical solution, especially for relatively thick plates, localized loads, contact-type loading, or cases with strong through-thickness constraints.

The simply supported boundary can be expressed by

$$\sigma_{x}^{i}=u_{y}^{i}=u_{z}^{i}=0,\;\mathrm{at}\;x\;=\;0,\;a$$

(5) $$\sigma_{y}^{i}=u_{x}^{i}=u_{z}^{i}=0,\;\mathrm{at}\;y\;=\;0,\;b$$

By using the state-space approach [27], the out-of-plane variables in the governing equations of Equations (1)–(3) can be rearranged into

(6) $$\frac{\partial }{\partial z}X^{i}(x,y,z,t)=MX^{i}(x,y,z,t),$$

where

$$X^{i}=\left[\begin{array}{c} u_{x}^{i}\\ u_{y}^{i}\\ u_{z}^{i}\\ \tau_{xz}^{i}\\ \tau_{yz}^{i}\\ \sigma_{z}^{i} \end{array}\right],\;M^{i}=\left[\begin{array}{cccccc} 0 & 0 & -\frac{\partial }{\partial x} & \frac{1}{G^{i}} & 0 & 0\\ 0 & 0 & -\frac{\partial }{\partial y} & 0 & \frac{1}{G^{i}} & 0\\ -\frac{\lambda^{i}}{\lambda^{i}+2G^{i}}\frac{\partial }{\partial x} & -\frac{\lambda^{i}}{\lambda^{i}+2G^{i}}\frac{\partial }{\partial y} & 0 & 0 & 0 & \frac{1}{\lambda^{i}+2G^{i}}\\ -\Phi^{i}\frac{\partial^{2}}{\partial x^{2}}-G^{i}\frac{\partial^{2}}{\partial y^{2}} & -L^{i}\frac{\partial^{2}}{\partial x\partial y} & 0 & 0 & 0 & -\frac{\lambda^{i}}{\lambda^{i}+2G^{i}}\frac{\partial }{\partial x}\\ -L^{i}\frac{\partial^{2}}{\partial x\partial y} & -\Phi^{i}\frac{\partial^{2}}{\partial y^{2}}-G^{i}\frac{\partial^{2}}{\partial x^{2}} & 0 & 0 & 0 & -\frac{\lambda^{i}}{\lambda^{i}+2G^{i}}\frac{\partial }{\partial y}\\ 0 & 0 & 0 & -\frac{\partial }{\partial x} & -\frac{\partial }{\partial y} & 0 \end{array}\right],$$

in which $\Phi^{i}=\lambda^{i}+2G^{i}-{\left(\lambda^{i}\right)}^{2}/(\lambda^{i}+2G^{i})$, $L^{i}=\lambda^{i}+G^{i}-{\left(\lambda^{i}\right)}^{2}/(\lambda^{i}+2G^{i})$. For simply supported boundaries, the out-of-plane stresses and displacements can be expanded in the following double Fourier series in the *x*- and *y*-directions

(7) $$\left[\begin{array}{c} u_{x}^{i}\\ u_{y}^{i}\\ u_{z}^{i}\\ \tau_{xz}^{i}\\ \tau_{yz}^{i}\\ \sigma_{z}^{i} \end{array}\right]=\sum_{m=1}^{\infty }\sum_{n=1}^{\infty }\left[\begin{array}{c} u_{x,mn}^{i}(z,t)\cos(\alpha_{m}x)\sin(\beta_{n}y)\\ u_{y,mn}^{i}(z,t)\sin(\alpha_{m}x)\cos(\beta_{n}y)\\ u_{z,mn}^{i}(z,t)\sin(\alpha_{m}x)\sin(\beta_{n}y)\\ \tau_{xz,mn}^{i}(z,t)\cos(\alpha_{m}x)\sin(\beta_{n}y)\\ \tau_{yz,mn}^{i}(z,t)\sin(\alpha_{m}x)\cos(\beta_{n}y)\\ \sigma_{z,mn}^{i}(z,t)\sin(\alpha_{m}x)\sin(\beta_{n}y) \end{array}\right],$$

where $\alpha_{m}=m\pi /a$ and $\beta_{n}=n\pi /b$. By substituting Equation (7) into Equation (6), one obtains

(8) $$\frac{d}{dz}X_{mn}^{i}(z,t)=D_{mn}^{i}X_{mn}^{i}(z,t),$$

where

$$D_{mn}^{i}=\left[\begin{array}{cccccc} 0 & 0 & -\alpha_{m} & \frac{1}{G^{i}} & 0 & 0\\ 0 & 0 & -\beta_{n} & 0 & \frac{1}{G^{i}} & 0\\ \frac{\lambda^{i}\alpha_{m}}{\lambda^{i}+2G^{i}} & \frac{\lambda^{i}\beta_{n}}{\lambda^{i}+2G^{i}} & 0 & 0 & 0 & \frac{1}{\lambda^{i}+2G^{i}}\\ \alpha_{m}^{2}\Phi^{i}+\beta_{n}^{2}G^{i} & \alpha_{m}\beta_{n}L^{i} & 0 & 0 & 0 & -\frac{\lambda^{i}\alpha_{m}}{\lambda^{i}+2G^{i}}\\ \alpha_{m}\beta_{n}L^{i} & \beta_{n}^{2}\Phi^{i}+\alpha_{m}^{2}G^{i} & 0 & 0 & 0 & -\frac{\lambda^{i}\beta_{n}}{\lambda^{i}+2G^{i}}\\ 0 & 0 & 0 & \alpha_{m} & \beta_{n} & 0 \end{array}\right],\;X_{mn}^{i}=\left[\begin{array}{c} u_{x,mn}^{i}\\ u_{y,mn}^{i}\\ u_{z,mn}^{i}\\ \tau_{xz,mn}^{i}\\ \tau_{yz,mn}^{i}\\ \sigma_{z,mn}^{i} \end{array}\right].$$

The solution of the state-space matrix equation of Equation (8) is as follows:

(9) $$X_{mn}^{i}(z,t)=e^{D_{mn}^{i}(z-d_{i-1})}X_{mn}^{i}(d_{i-1},t),$$

where $d_{i}=\sum_{i=1}^{p}h_{i}$. Using Equations (1) and (2), other in-plane variables can be expressed in terms of the out-of-plane variables as

$$\sigma_{x}^{i}=\sum_{m=1}^{\infty }\sum_{n=1}^{\infty }\left[-\alpha_{m}\Phi^{i}u_{x,mn}^{i}-\beta_{n}(L^{i}-G^{i})u_{y,mn}^{i}+\frac{\lambda^{i}}{\lambda^{i}+2G^{i}}\sigma_{z,mn}^{i}\right]\sin(\alpha_{m}x)\sin(\beta_{n}y),$$

$$\sigma_{y}^{i}=\sum_{m=1}^{\infty }\sum_{n=1}^{\infty }\left[-\alpha_{m}(L^{i}-G^{i})u_{x,mn}^{i}-\beta_{n}\Phi^{i}u_{y,mn}^{i}+\frac{\lambda^{i}}{\lambda^{i}+2G^{i}}\sigma_{z,mn}^{i}\right]\sin(\alpha_{m}x)\sin(\beta_{n}y),$$

(10) $$\tau_{xy}^{i}=\sum_{m=1}^{\infty }\sum_{n=1}^{\infty }G^{i}\left[\beta_{n}u_{x,mn}^{i}+\alpha_{m}u_{y,mn}^{i}\right]\cos(\alpha_{m}x)\cos(\beta_{n}y).$$

### 2.3. Equations for an Interlayer

For the interlayers in the LG plate, the generalized Maxwell model is adopted to characterize their viscoelastic behavior, while the temperature effect on viscoelasticity is described using the WLF equation [28,29]. The WLF equation is introduced based on the time–temperature superposition principle. The polymer interlayer is assumed to be thermorheologically simple, so temperature only shifts the relaxation curve along the logarithmic time axis without changing its shape. Accordingly, the relaxation behavior at different temperatures can be described by a temperature shift factor. This assumption is limited to the calibrated range of the master curve, and thermorheologically complex effects such as aging or curing are not considered. In this study, all polymer interlayers are assumed to be made of the same material; therefore, the layer index is omitted in the following viscoelastic constitutive description. Thus, the shear relaxation modulus $G^{*}(t)$ and the Young’s relaxation modulus $E^{*}(t)$ can be described by

(11) $$G^{*}(t)=G_{\infty }^{*}+\sum_{j=1}^{K}G_{j}^{*}e^{-t/\delta^{*}\theta_{j}^{*}}=G_{0}^{*}-\sum_{j=1}^{K}G_{j}^{*}(1-e^{-t/\delta^{*}\theta_{j}^{*}}),\;E^{*}(t)=2(1+\mu^{*})G^{*}(t),$$

(12) $$\log_{10}\delta^{*}=-\frac{C_{1}^{*}(T-T_{ref}^{*})}{C_{2}^{*}+(T-T_{ref}^{*})}.$$

where the superscript * denotes variables associated with the interlayer, $G_{\infty }^{*}$ is the long-term shear modulus, $G_{j}^{*}$ and $\theta_{j}^{*}$ are the modulus and relaxation time of the *j*-th Maxwell branch, respectively, $G_{0}^{*}$ is given by $G_{\infty }^{*}+\sum_{j=1}^{K}G_{j}^{*}$, and $\delta^{*}$ is the temperature shift factor characterizing the shift in the viscoelastic time scale caused by temperature variation, *T* is the current or analysis temperature, $T_{ref}^{*}$ is the reference temperature at which the master curve or Prony-series parameters are defined, $C_{1}^{*}$ and $C_{2}^{*}$ are empirical constants usually determined by fitting experimental data.

For the stress and deformation of the interlayer, two treatments are adopted here: (1) treating the interlayer through interface conditions, which requires the glass layer thickness to be much larger than the interlayer thickness and (2) modeling the interlayer as an individual layer.

First, the formulation based on treating the interlayer through interface conditions is established. According to the Boltzmann superposition principle [30], the constitutive equations of the *k*-th (*k* = 1, 2, …, *p* − 1) viscoelastic interlayer are formulated in the following convolution form:

$$\tau_{xz}^{*,k}(x,y,z,t)=\int_{-\infty }^{t}G^{*}(t-\xi )\frac{\partial \gamma_{xz}^{*,k}(x,y,z,\xi )}{\partial \xi }d\xi ,$$

$$\tau_{yz}^{*,k}(x,y,z,t)=\int_{-\infty }^{t}G^{*}(t-\xi )\frac{\partial \gamma_{yz}^{*,k}(x,y,z,\xi )}{\partial \xi }d\xi ,$$

(13) $$\sigma_{z}^{*,k}(x,y,z,t)=\int_{-\infty }^{t}E^{*}(t-\xi )\frac{\partial \varepsilon_{z}^{*,k}(x,y,z,\xi )}{\partial \xi }d\xi ,$$

Equation (13) indicates that the interlayer stresses at time *t* depend on the complete strain history. Since the glass layer thickness is much larger than the interlayer thickness, the interlayer strains are expressed by

(14) $$\gamma_{xz}^{*,k}=\frac{u^{i+1}-u^{i}}{\Delta h},\;\gamma_{yz}^{*,k}=\frac{v^{i+1}-v^{i}}{\Delta h},\;\varepsilon_{z}^{*,k}=\frac{w^{i+1}-w^{i}}{\Delta h},\;(i\;=\;k,\;z\;=\;d_{i}).$$

Meanwhile, traction continuity at the glass-interlayer interfaces gives

(15) $$\sigma_{z}^{i}=\sigma_{z}^{*,k}=\sigma_{z}^{i+1},\;\tau_{xz}^{i}=\tau_{xz}^{*,k}=\tau_{xz}^{i+1},\;\tau_{yz}^{i}=\tau_{yz}^{*,k}=\tau_{yz}^{i+1},\;(i\;=\;k,\;z\;=\;d_{i})$$

The matrix form of Equations (13)–(15), followed by the Laplace transform, is given by

(16) $${\overset{⌢}{X}}_{mn}^{i+1}(d_{i},s)=K_{mn}(s){\overset{⌢}{X}}_{mn}^{i}(d_{i},s),$$

where

$$K_{mn}(s)=\left[\begin{array}{cccccc} 1 & 0 & 0 & \frac{\Delta h}{s{\overset{⌢}{G}}^{*}(s)} & 0 & 0\\ 0 & 1 & 0 & 0 & \frac{\Delta h}{s{\overset{⌢}{G}}^{*}(s)} & 0\\ 0 & 0 & 1 & 0 & 0 & \frac{\Delta h}{s{\overset{⌢}{E}}^{*}(s)}\\ 0 & 0 & 0 & 1 & 0 & 0\\ 0 & 0 & 0 & 0 & 1 & 0\\ 0 & 0 & 0 & 0 & 0 & 1 \end{array}\right].$$

Subsequently, the formulation in which the interlayer is modeled as an individual layer is established. In this case, the governing equations of the interlayer are similar to those of the glass layers. The difference is that the former is a viscoelastic material, whereas the latter is purely elastic. Therefore, referring to Equation (8), the general solutions for the stresses and displacements of the interlayer in the Laplace domain can be expressed as follows:

(17) $${\overset{⌢}{X}}_{mn}^{i}(z,s)=e^{D_{mn}^{i}(z-d_{k-1})}{\overset{⌢}{X}}_{mn}^{i}(d_{i-1},s),$$

where

$$D_{mn}^{i}=\left[\begin{array}{cccccc} 0 & 0 & -\alpha_{m} & \frac{1}{s{\overset{⌢}{G}}^{*}(s)} & 0 & 0\\ 0 & 0 & -\beta_{n} & 0 & \frac{1}{s{\overset{⌢}{G}}^{*}(s)} & 0\\ \frac{s\overset{⌢}{\lambda }(s)\alpha_{m}}{s\overset{⌢}{\lambda }(s)+2s{\overset{⌢}{G}}^{*}(s)} & \frac{s\overset{⌢}{\lambda }(s)\beta_{n}}{s\overset{⌢}{\lambda }(s)+2s{\overset{⌢}{G}}^{*}(s)} & 0 & 0 & 0 & \frac{1}{s\overset{⌢}{\lambda }(s)+2s{\overset{⌢}{G}}^{*}(s)}\\ \alpha_{m}^{2}s{\overset{⌢}{\Phi }}^{i}(s)+\beta_{n}^{2}s{\overset{⌢}{G}}^{*}(s) & \alpha_{m}\beta_{n}s\overset{⌢}{L^{i}}(s) & 0 & 0 & 0 & -\frac{s\overset{⌢}{\lambda }(s)\alpha_{m}}{s\overset{⌢}{\lambda }(s)+2s{\overset{⌢}{G}}^{*}(s)}\\ \alpha_{m}\beta_{n}s\overset{⌢}{L^{i}}(s) & \beta_{n}^{2}s{\overset{⌢}{\Phi }}^{i}(s)+\alpha_{m}^{2}s{\overset{⌢}{G}}^{*}(s) & 0 & 0 & 0 & -\frac{s\overset{⌢}{\lambda }(s)\beta_{n}}{s\overset{⌢}{\lambda }(s)+2s{\overset{⌢}{G}}^{*}(s)}\\ 0 & 0 & 0 & \alpha_{m} & \beta_{n} & 0 \end{array}\right]$$

### 2.4. Analytical Solution of Stresses and Displacements

For the two treatment schemes described above, combining Equations (9) and (16), and combining Equations (16) and (17), respectively, both yield the relationships between the stresses and displacements of the *i*-th glass layer and those of the first glass layer in the Laplace domain:

(18) $${\overset{⌢}{X}}_{mn}^{i}(z,s)=e^{D_{mn}^{i}(z-d_{i-1})}\prod_{j=i-1}^{1}\left[K_{mn}(s)e^{D_{mn}^{j}h_{j}}\right]{\overset{⌢}{X}}_{mn}^{1}(0,s),$$

in which the matrix $K_{mn}(s)$ reduces to the identity matrix for the case in which the interlayer is modeled as an individual layer. By setting *i* = *p* and *z* = *H* in the above equation, the relationship between the upper and lower surfaces of the LG plate can be obtained as follows:

(19) $$\left[\begin{array}{c} {\overset{⌢}{u}}_{x,mn}^{p}(H,s)\\ {\overset{⌢}{u}}_{y,mn}^{p}(H,s)\\ {\overset{⌢}{u}}_{z,mn}^{p}(H,s)\\ {\overset{⌢}{\tau }}_{xz,mn}^{p}(H,s)\\ {\overset{⌢}{\tau }}_{yz,mn}^{p}(H,s)\\ {\overset{⌢}{\sigma }}_{z,mn}^{p}(H,s) \end{array}\right]=\left[\begin{array}{cc} S_{mn}^{11} & S_{mn}^{12}\\ S_{mn}^{21} & S_{mn}^{22} \end{array}\right]\left[\begin{array}{c} {\overset{⌢}{u}}_{x,mn}^{1}(0,s)\\ {\overset{⌢}{u}}_{y,mn}^{1}(0,s)\\ {\overset{⌢}{u}}_{z,mn}^{1}(0,s)\\ {\overset{⌢}{\tau }}_{xz,mn}^{1}(0,s)\\ {\overset{⌢}{\tau }}_{yz,mn}^{1}(0,s)\\ {\overset{⌢}{\sigma }}_{z,mn}^{1}(0,s) \end{array}\right],$$

where $S_{mn}=e^{D_{mn}^{i}h_{i}}\prod_{j=i-1}^{1}\left[K_{mn}(s)e^{D_{mn}^{j}h_{j}}\right]$ and $S_{mn}^{**}$ are the corresponding sub-matrices. The known loading conditions on the upper and lower surfaces of the LG plate are as follows:

$${\overset{⌢}{\sigma }}_{z,mn}^{1}(0,s)={\overset{⌢}{\tau }}_{xz,mn}^{1}(0,s)={\overset{⌢}{\tau }}_{yz,mn}^{1}(0,s)=0,$$

(20) $${\overset{⌢}{\sigma }}_{z,mn}^{p}(H,s)=-\frac{1}{s}q_{mn},\;{\overset{⌢}{\tau }}_{xz,mn}^{p}(H,s)={\overset{⌢}{\tau }}_{yz,mn}^{p}(H,s)=0.$$

where $q_{mn}=\frac{4}{ab}\int_{0}^{a}\int_{0}^{b}q(x,y)\sin(\alpha_{m}x)\sin(\beta_{n}y)dxdy$. Substitution of Equation (20) into Equation (19) yields the following:

(21) $$\left[\begin{array}{c} {\overset{⌢}{u}}_{x,mn}^{1}(0,s)\\ {\overset{⌢}{u}}_{y,mn}^{1}(0,s)\\ {\overset{⌢}{u}}_{z,mn}^{1}(0,s) \end{array}\right]={\left[S_{mn}^{21}(s)\right]}^{-1}\left[\begin{array}{c} 0\\ 0\\ -q_{mn}/s \end{array}\right],$$

Once ${\overset{⌢}{X}}_{mn}^{1}(0,s)$ is determined, the analytical solutions for the stresses and displacements of the *i*-th layer in the Laplace domain, i.e., ${\overset{⌢}{X}}_{mn}^{i}(z,s)$, are obtained by substituting Equation (21) into Equation (18).

By combining Cramer’s rule with the residue theorem, the *k*-th element of column vector ${\overset{⌢}{X}}_{mn}^{i}(z,s)$ can be expressed as follows:

(22) $${\overset{⌢}{X}}_{mn}^{i,k}(z,s)=\frac{\sum_{\lambda =0}^{\zeta }\omega_{mn}^{ik\lambda }s^{\lambda }}{\sum_{\lambda =0}^{\zeta }\eta_{mn}^{i\lambda }s^{\lambda +1}}\frac{q_{mn}}{s}=\sum_{\beta =1}^{\zeta +1}\frac{c_{\beta mn}^{ik}}{s-s_{mn}^{i\beta }}\frac{q_{mn}}{s},$$

where

$$\omega_{mn}^{ik\lambda }=\underset{s\to +\infty }{\lim}\frac{1}{s^{\lambda }}\left(\left|T_{mn}^{i,k}(s)\right|-\sum_{a=\lambda +1}^{\zeta }\omega_{mn}^{ika}s^{a}\right),\;\eta_{mn}^{i\lambda }=\underset{s\to +\infty }{\lim}\frac{1}{s^{\lambda }}\left(\left|T_{mn}^{i}(s)\right|-\sum_{a=\lambda +1}^{\zeta }\eta_{mn}^{ia}s^{a}\right),$$

$$c_{\beta mn}^{ik}=\frac{\sum_{\lambda =0}^{\zeta }\omega_{mn}^{ik\lambda }{\left(s_{mn}^{i\beta }\right)}^{\lambda }}{\sum_{\lambda =0}^{\zeta }(\lambda +1)\eta_{mn}^{i\lambda }{\left(s_{mn}^{i\beta }\right)}^{\lambda }},\;T_{mn}^{i}(s)=e^{D_{mn}^{i}(z-d_{i-1})}\prod_{j=i-1}^{1}\left[K_{mn}(s)e^{D_{mn}^{j}h_{j}}\right],$$

in which,$T_{mn}^{i,k}(s)$ denotes the matrix obtained by replacing the *k*-th column of ${\overset{⌢}{X}}_{mn}^{1}(0,s)$, and $s_{mn}^{i\beta }$ are the roots of the characteristic equation $\sum_{\lambda =0}^{\zeta }\eta_{mn}^{i\lambda }s^{\lambda +1}=0$. The characteristic roots in Equation (22) are obtained from the zeros of the determinant in the denominator of the Laplace-domain solution. Specifically, the determinant is first expressed as a scalar characteristic function of the Laplace variable, and its roots are computed using a complex root-search procedure. The obtained roots are then substituted back into the characteristic function to check the residual accuracy before residue evaluation. Numerical stability is ensured through root filtering and tolerance control. Roots with positive real parts are excluded because they would lead to non-physical growth in the time-domain response. Roots with sufficiently small imaginary parts are treated as real roots, and nearly repeated roots are merged according to a prescribed tolerance to avoid duplicated residue contributions.

The analytical solutions for the out-of-plane stresses and displacements of the LG plate in the time domain are obtained by applying the inverse Laplace transform to the above expressions:

(23) $$X_{mn}^{i,k}(z,t)=\sum_{\beta =1}^{\zeta +1}\sum_{j=1}^{g}c_{\beta mn}^{ik}e^{-s_{mn}^{i\beta }t}q_{mn}H(t),$$

where *H*(*t*) denotes the Heaviside step function.

### 2.5. Extension of the Present Model

The analytical model developed above is based on the small-deformation theory. For cases involving geometrically nonlinear deformation, the present formulation can be extended to a von Kármán-type framework by replacing the linear in-plane strain components with nonlinear strain-displacement relations. Accordingly, the following geometric relations are introduced:

(24) $$\varepsilon_{x}=\frac{\partial u_{x}}{\partial x}+\frac{1}{2}{\left(\frac{\partial u_{z}}{\partial x}\right)}^{2},\;\varepsilon_{y}=\frac{\partial u_{y}}{\partial y}+\frac{1}{2}{\left(\frac{\partial u_{z}}{\partial y}\right)}^{2},\;\gamma_{xy}=\frac{\partial u_{x}}{\partial y}+\frac{\partial u_{y}}{\partial x}+\frac{\partial u_{z}}{\partial x}\frac{\partial u_{z}}{\partial y},$$

The additional quadratic terms involving the transverse displacement gradients account for the membrane stretching induced by moderately large transverse deflection. The resulting nonlinear governing equations may be solved using an incremental-iterative procedure.

Although a uniform temperature is adopted in the present formulation, the model can be further extended to consider a non-uniform steady temperature field through the thickness direction of the LG plate. For this purpose, a one-dimensional steady heat conduction model is introduced. The thickness of the *i*-th layer is denoted by *h_i_* = *z_i_* − *z_i_*_−1_. Assuming constant thermal conductivity within each layer, the governing equation can be written as follows:

(25) $$k_{i}\frac{d^{2}T_{i}}{dz^{2}}+Q_{i}=0,$$

Here, *T_i_*(*z*) is the temperature of the *i*-th layer, *k_i_* is the thermal conductivity and *Q_i_* is the equivalent volumetric heat source induced by solar radiation. The solar radiation absorbed by the glass layer is simplified as a uniformly distributed heat source,

(26) $$Q_{i}=\frac{\eta_{i}I_{s}}{h_{i}},$$

where *I_s_* is the incident solar radiation intensity and *η_i_* is the solar absorption ratio of the *i*-th layer. For transparent polymer interlayers, the absorption of solar radiation is neglected, and therefore, *Q_i_* = 0. The temperature distribution in the *i*-th layer can then be expressed as

(27) $$T_{i}(z)=-\frac{Q_{i}}{2k_{i}}{\left(z-z_{i-1}\right)}^{2}+A_{i}(z-z_{i-1})+B_{i},$$

where *A_i_* and *B_i_* are unknown constants determined from the boundary and interface conditions. At the outdoor surface, the convective boundary condition is given by

(28) $$-k_{1}{\left.\frac{dT_{1}}{dz}\right|}_{z=0}=h_{out}\left[T_{out}-T_{1}(0)\right],$$

where *T_out_* is the outdoor air temperature and *h_out_* is the outdoor convective heat transfer coefficient. At the indoor surface, the convective boundary condition is written as

(29) $$-\lambda_{p}{\left.\frac{dT_{p}}{dz}\right|}_{z=H}=h_{in}\left[T_{p}(H)-T_{in}\right],$$

where *T_in_* is the indoor air temperature, *h_in_* is the indoor convective heat transfer coefficient, *p* is the total number of layers, and *H* is the total thickness of the laminated glass plate. At each interface between adjacent layers, the temperature and heat flux are continuous:

(30) $$T_{i}(z_{i})=T_{i+1}(z_{i}),$$

(31) $$k_{i}{\left.\frac{dT_{i}}{dz}\right|}_{z=z_{i}}=k_{i+1}{\left.\frac{dT_{i+1}}{dz}\right|}_{z=z_{i}},\;i=1,2,\ldots ,p-1,$$

By solving the above linear algebraic equations, the constants *A*_i_ and *B*_i_ can be obtained, and the steady temperature distribution through the thickness of the multilayer laminated glass plate is determined. In the present study, this temperature field is used as an environmental variable for evaluating the temperature-dependent viscoelastic properties of the polymer interlayers, while thermally induced stresses in the glass layers are not considered.

For post-fracture applications, the present formulation may be further extended by introducing a fragmented-glass representation. In this approach, the fractured glass layer can be represented by discrete glass fragments, while the polymer interlayer is retained to transfer shear and maintain the integrity of the fragmented glass system. The reduced stiffness of the fractured glass and the fragment size distribution should be calibrated from post-breakage tests.

Environmental aging and hygrothermal exposure may further affect the long-term response of polymer interlayers. Ultraviolet radiation, humidity, thermal cycling, and plasticizer migration can change the relaxation modulus, relaxation time, and WLF constants of the interlayer. To incorporate these effects, an aging variable *χ* may be introduced to describe the cumulative environmental exposure. Accordingly, the shear relaxation modulus can be extended as

(32) $$G^{*}(t,T,\chi )=G_{\infty }^{*}(\chi )+\sum_{j=1}^{N}G_{j}^{*}(\chi )e^{-t/\theta_{j}(T,\chi )},$$

where $G_{\infty }^{*}(\chi )$, $G_{j}^{*}(\chi )$, and $\theta_{j}^{*}(T,\chi )$ are aging-dependent viscoelastic parameters. The temperature shift factor in the WLF equation may also be written as an aging-dependent function,

(33) $$\log a_{T}(T,\chi )=-\frac{C_{1}^{*}(\chi )(T-T_{\mathrm{ref}})}{C_{2}^{*}(\chi )+(T-T_{\mathrm{ref}})},$$

where $C_{1}^{*}(\chi )$ and $C_{2}^{*}(\chi )$ denote the aging-dependent WLF constants. These material functions should be calibrated through accelerated aging tests followed by DMA, creep, relaxation, or shear tests. In the present study, these aging effects are not considered, but the above formulation provides a possible extension for future long-term durability analysis of laminated glass plates.

The present model may also be extended to electroactive or electrothermally activated dual-curing nanocomposite interlayers [31,32]. Conductive nanofillers embedded in such interlayers may generate Joule heating or self-heating under an applied electric field, thereby changing the local interlayer temperature and the viscoelastic relaxation process. Within the present framework, when the electrothermal field is mainly distributed through the thickness, the interlayer can be divided into several thin sublayers, each assigned temperature- and curing-dependent viscoelastic properties, and the corresponding transfer matrices can be assembled following the multilayer formulation. If the shear modulus varies simultaneously in space and time, the model can be further implemented through an incremental thermo-viscoelastic scheme, in which the electrothermal field and local material parameters are updated at each time step before recalculating the mechanical response.

## 3. Numerical Examples

In this section, numerical analyses are conducted to investigate the long-term bending behavior of the LG plate with temperature-dependent viscoelastic interlayers. Unless otherwise specified, the studied LG plate considered herein consists of two glass layers and one polymer interlayer. The default parameters are taken as: *a* = *b* = 3000 mm, *E*_1_ = *E*_2_ = 70,000 MPa, *ν*_1_ = *ν*_2_ = 0.3, *h*_1_ = *h*_2_ = 10 mm, *T*_ref_ = 20 °C, *q* = 1.5sin(π*x*/*a*) sin(π*y*/*b*) × 10^−3^ N/mm^2^. Three representative polymer interlayer materials are considered. Their WLF constants and the corresponding viscoelastic Prony-series parameters are given in Table 1 and Table 2 [15]. In the following analyses, the stress components and displacement are evaluated at selected representative locations, namely

$$\begin{array}{c} \sigma_{x}\;\mathrm{at}\;(x,\;y,\;z)=(0.5a,0.5b,0),\;\tau_{xy}\;\mathrm{at}\;(x,\;y,\;z)\;=\;(0,\;0,\;0),\\ \tau_{xz}\;\mathrm{at}\;(x,\;y,\;z)=(0,0.5b,h_{1}),\;u_{z}\;\mathrm{at}\;(x,\;y,\;z)\;=\;(0.5a,0.5b,0). \end{array}$$

### 3.1. Convergence Analysis

Since the present solution is expressed as a double Fourier series, finite truncation is required for numerical implementation. The series terms in the *x*- and *y*-directions are retained as *m*, *n* = 1, 2, …, *N*. To assess the truncation effect, *N* = 1, 3, 5, 7 and 9 are examined at *t* = 10^3^, 10^6^, and 10^9^ s, which represent different stages of the viscoelastic response. The absolute values of the selected stress and displacement components are listed in Table 3.

It can be seen from Table 3 that the results tend to converge rapidly as the truncation number increases. The variation of the calculated responses becomes very small when *N* is increased from 7 to 9, indicating that sufficient convergence accuracy has been achieved. Therefore, *N* = 9 is adopted as the truncation number for the series solution in the subsequent analyses, unless stated otherwise.

### 3.2. Validation Analysis

To further evaluate the applicability of the present model to long-term viscoelastic behavior, a comparison is made with the experimental results reported by Centelles et al. [16]. The tested laminated glass plates consisted of two 8 mm tempered glass layers and a 1.52 mm PVB or SentryGlas Plus (SGP) interlayer. The plate size is 1580 mm × 1000 mm, with a support span of 1500 mm. A uniformly distributed water load of 3 × 10^−3^ N/mm^2^ is applied at 23 °C for approximately four months. Only the loading and creep stage is considered here, and the central deflection is compared.

**Figure 2:**
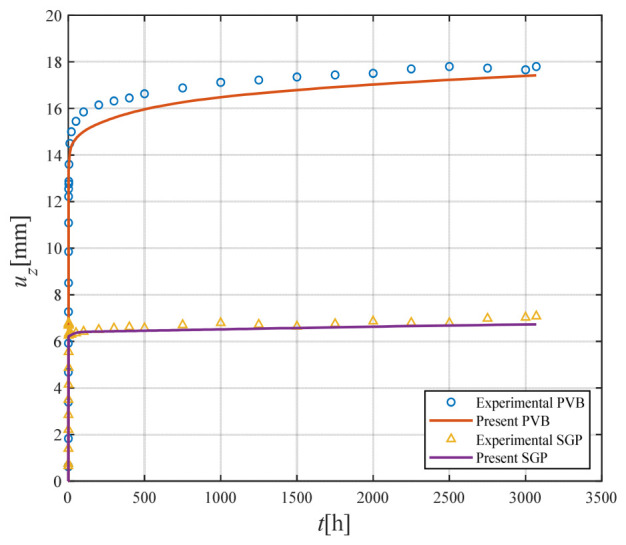
Comparison of mid-span deflection histories between the present model and experimental results for PVB and SGP LG plates.

As shown in Figure 2, the central deflection histories predicted by the present model are compared with the experimental data for both PVB LG and SGP LG plates. Based on the experimental data before the end of the long-term sustained loading stage, the maximum absolute errors are 6.15% and 8.30% for the PVB LG and SGP LG plates, respectively. These results indicate that the present formulation can reasonably predict the long-term deflection response of laminated glass plates with different viscoelastic interlayers.

The accuracy of the present solution is evaluated by comparison with an in-house Kirchhoff–Love (KL) plate reference solution and an FE solution. The KL reference solution, in which transverse shear deformation is neglected, was developed in Appendix A, in reference to the analytical study of Li et al. [33] on laminated functionally graded beams with a viscoelastic interlayer. The FE model is established in ANSYS (version: 2023) using SOLSH190 solid-shell elements, where the glass layers are modeled as linear elastic layers and the PVB interlayer is defined using the same Prony-series viscoelastic parameters as those in the present solution. For the FE mesh discretization, the plate is divided into 60 × 60 elements in the in-plane directions, with one element through the thickness of each physical layer, resulting in 10,800 elements, 14,884 nodes, and 43,689 active equations. Table 4 gives the comparisons of *σ_x_*, *τ_xz_* and *u_z_* among the present solution, KL solution, and FE solution for different length-to-thickness ratios *a*/*H* at *t* = 10^9^ s. As shown in Table 4, the present results agree well with the FE results because both methods are based on 3D elasticity theory. Compared with the FE method, which requires spatial meshing and step-by-step time integration, the present analytical solution can directly evaluate the response at any specified time, thereby offering higher computational efficiency. The discrepancy between the present solution and the KL solution becomes more pronounced when *a*/*H* is small, particularly in the displacement response. With increasing *a*/*H*, the KL results gradually converge to the present results. This observation indicates that transverse shear effects are more significant in relatively thick plates, whereas the KL theory becomes more applicable to thin plates. Beyond the accuracy comparison, the computational efficiency of the present solution is further evaluated using the LG plate with *a*/*H* = 100. For *t* = 10^3^, 10^6^, and 10^9^ s, the FE analysis is performed through 81, 111, and 141 sequential time steps, respectively, whereas the present solution directly evaluates the corresponding specified time points without spatial meshing. As shown in Table 5, the present solution requires much less CPU time and memory than the FE simulation. Even for *t* = 10^9^ s, the present solution is about 1.13 × 10^3^ times faster than the FE simulation, while its memory usage is only about 10.88% of that required by the FE model. This is because the FE method advances the solution step by step in time, where the response at each time step depends on the solution obtained from the preceding step.

In addition, the interface conditions model is compared with the individual layer model to clarify the influence of interlayer modeling, as shown in Table 6. The discrepancy decreases with decreasing interlayer thickness ratio and increasing time. This is because the interlayer gradually softens with viscoelastic relaxation, reducing the difference between the two modeling approaches. For Δ*h*/*h*_1_ = 0.152, the maximum error decreases from 20.36% at *t* = 10^6^ s to 9.665% at *t* = 10^9^ s, while for Δ*h*/*h*_1_ = 0.038, it decreases from 3.154% to 0.8142%. These results indicate that the interface conditions model is acceptable for thin interlayers and long-term responses, whereas the individual layer model better captures the through-thickness deformation and stress transfer for relatively thick interlayers. Therefore, the individual layer model is adopted in the following analysis.

### 3.3. Effects of Interlayer Thickness, Temperature and Material

**Figure 3:**
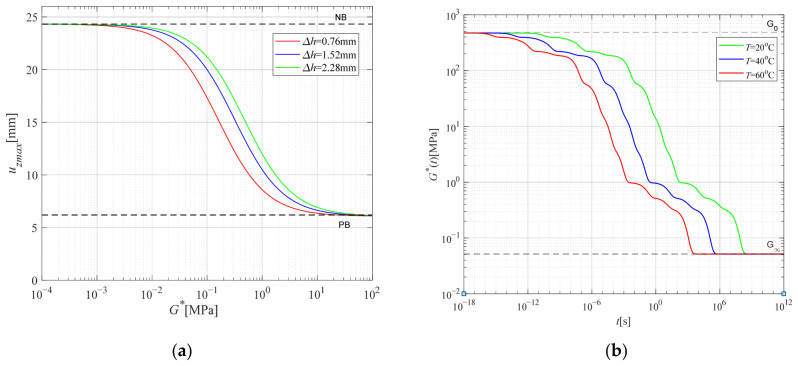
Effects of interlayer thickness and temperature on the maximum displacement and effective shear modulus of the PVB LG plate. (**a**) Effect of interlayer G* on *u_zmax_*; (**b**) Degradation of G*(*t*).

**Figure 4:**
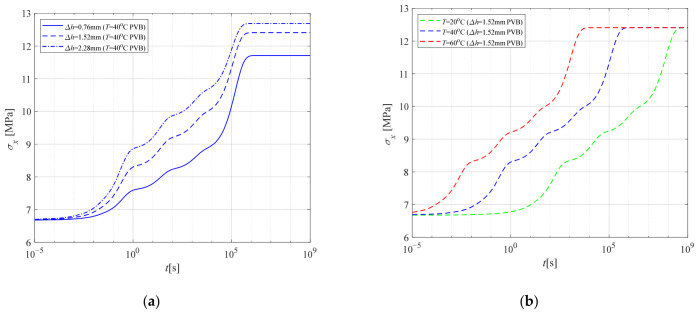

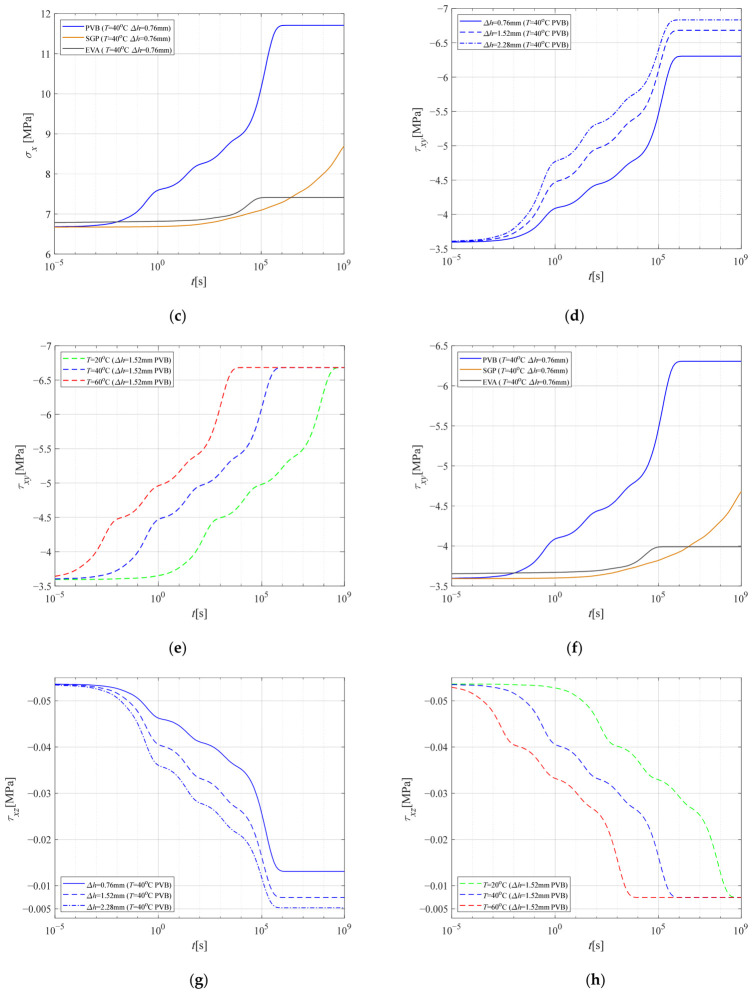

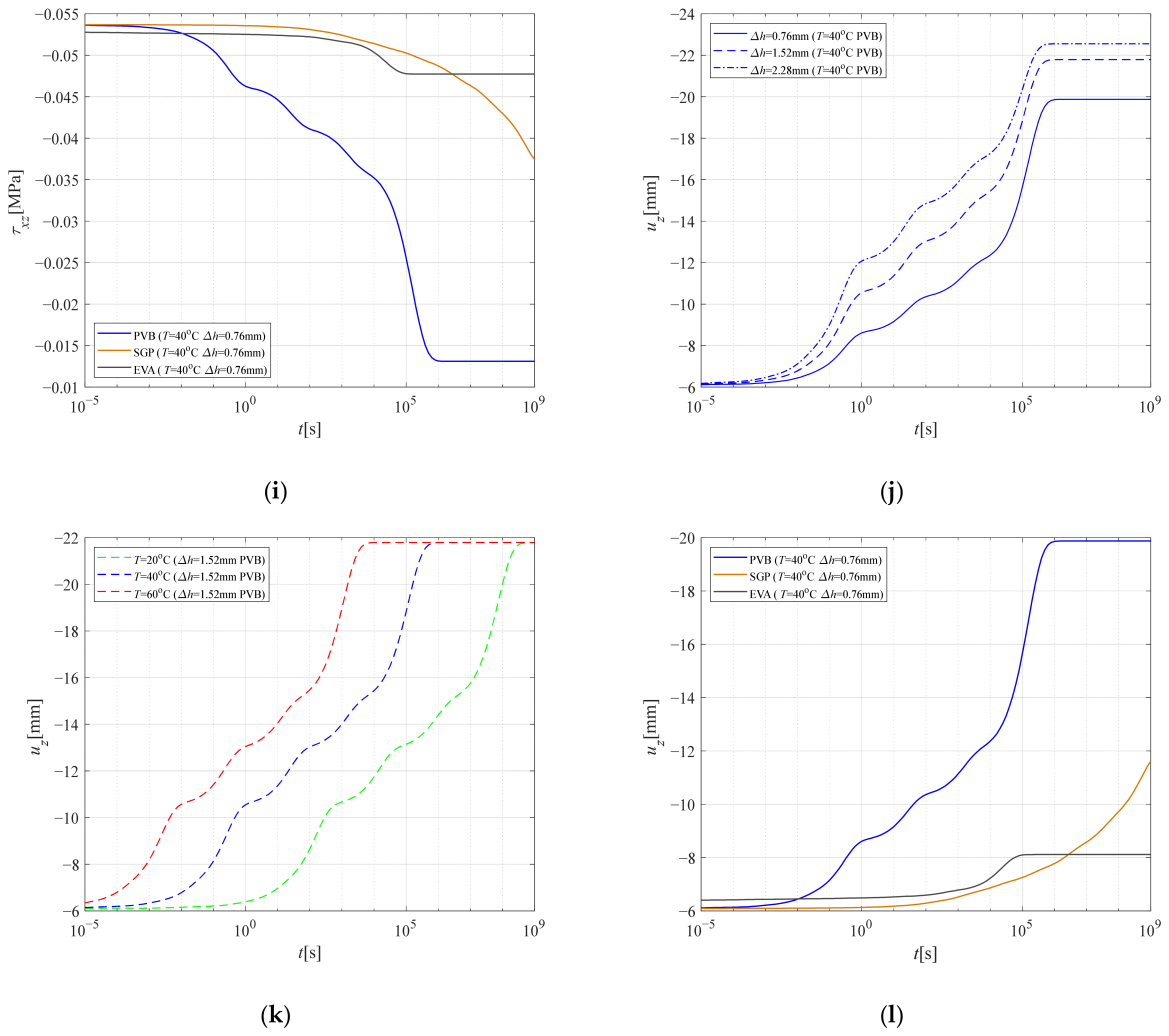
Effects of interlayer thicknesses of 0.76, 1.52, and 2.28 mm, temperatures of 20, 40, and 60 °C, and interlayer materials of PVB, SGP, and EVA on the time-varying stresses and displacement responses of LG plates. (**a**) *σ_x_* varying with *t* at different Δ*h*; (**b**) *σ_x_* varying with *t* at different *T*; (**c**) *σ_x_* varying with *t* for different materials; (**d**) *τ_xy_* varying with *t* at different Δ*h*; (**e**) *τ_xy_* varying with *t* at different *T*; (**f**) *τ_xy_* varying with *t* for different materials; (**g**) *τ_xz_* varying with *t* at different Δ*h;* (**h**) *τ_xz_* varying with *t* at different *T*; (**i**) *τ_xz_* varying with *t* for different materials; (**j**) *u_z_* varying with *t* at different Δ*h*; (**k**) *u_z_* varying with *t* at different *T*; (**l**) *u_z_* varying with *t* for different materials.

**Figure 5:**
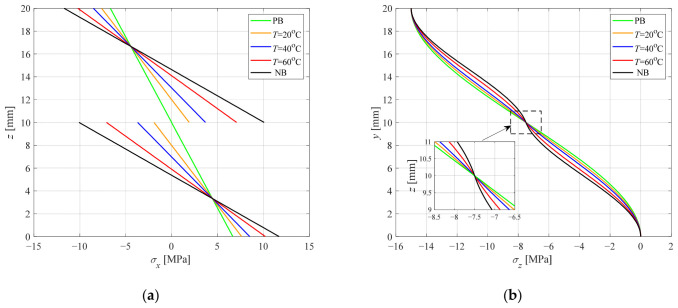

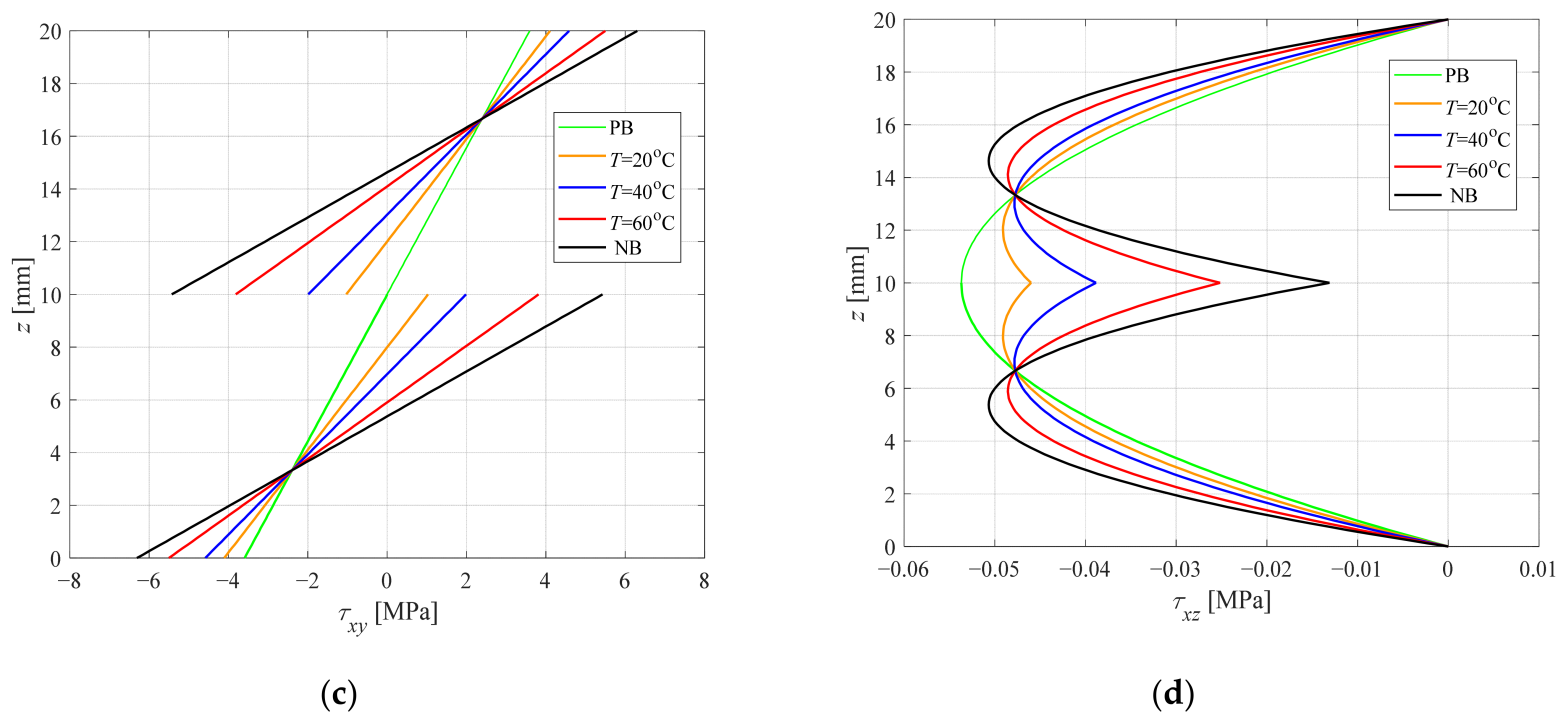
Distributions of through-thickness stresses in the PVB LG plate at temperatures of 20, 40, and 60 °C for Δ*h* = 0.76 mm and *t* = 10^3^ s. (**a**) *σ_x_* at *x* = 1500 mm, *y* = 1500 mm; (**b**) *σ_z_* at *x* = 1500 mm, *y* = 1500 mm; (**c**) *τ_xy_* at *x* = 0, *y* = 0; (**d**) *τ_xz_* at *x* = 0, *y* = 1500 mm.

The effects of interlayer thickness, temperature and material type on the long-term stresses and displacement responses of two-layer LG plates are investigated in this section. Figure 3 illustrates the relationship between the interlayer shear relaxation modulus, the maximum displacement and the time-dependent degradation of G*(*t*). Figure 4 further compares the time-dependent responses of LG plates with PVB, SGP and Ethylene-Vinyl Acetate (EVA) interlayers at *T* = 40 °C and Δ*h* = 0.76 mm. Meanwhile, Figure 5 presents the through-thickness stress distributions of the PVB LG plate for Δ*h* = 0.76 mm at *t* = 10^3^ s under *T* = 20, 40 and 60 °C.

It can be found from Figure 3 that (i) as the interlayer shear relaxation modulus G*(*t*) approaches a very small value, the adjacent glass layers behave as nearly unbonded layers, corresponding to the no-bonding (NB) case; consequently, the maximum deflection *u_zmax_* remains almost unchanged. As the interlayer shear modulus increases, the deflection decreases rapidly and then gradually approaches the perfect-bonding (PB) case. (ii) With increasing time, the shear modulus of the PVB interlayer gradually relaxes and eventually approaches a stable long-term value. This behavior is attributed to the stress relaxation of the viscoelastic interlayer under sustained loading, which leads to a progressive reduction in the interfacial shear-transfer capacity. Elevated temperature further accelerates this relaxation process according to the time-temperature superposition principle.

It can be found from Figure 4 that (i) with increasing time, the magnitudes of *σ_x_*, *τ_xy_* and *u_z_* increase gradually, while the magnitude of *τ_xz_* decreases. These response quantities ultimately converge to their corresponding NB solutions. This behavior agrees well with Figure 3a, indicating that the time-dependent reduction in the effective interlayer shear modulus weakens the composite action of the laminated plate and consequently results in a larger deflection. (ii) For the cases of 20, 40, and 60 °C, higher temperature leads to earlier stabilization of *σ_x_*, *τ_xy_*, *u_z_* and *τ_xz_* at their corresponding long-term values. This demonstrates that elevated temperature accelerates the relaxation process of the interlayer. Nevertheless, temperature primarily affects the time needed to reach the long-term state, while having only a limited effect on the final long-term stress and displacement responses. (iii) Similar to the effect of temperature, increasing the interlayer thickness also causes the stress and displacement responses to reach their long-term values earlier at the same temperature, although this acceleration effect is relatively limited. More importantly, increasing the interlayer thickness increases the long-term values of *σ_x_*, *τ_xy_* and *u_z_* while reducing the long-term value of *τ_xz_*. This is because the interfacial shear stiffness decreases with increasing Δ*h*, which weakens the constraint provided by the interlayer and consequently increases the long-term values of *σ_x_*, *τ_xy_* and *u_z_*. (iv) The stress and displacement responses exhibit a delayed evolution compared with the relaxation of the shear modulus. This time-lag effect is associated with the intrinsic memory behavior of viscoelastic materials. (v) The interlayer material has a pronounced influence on the long-term stress and displacement responses. PVB exhibits the largest long-term values of *σ_x_*, *τ_xy_* and *u_z_*, together with the smallest long-term value of *τ_xz_*. This indicates a greater long-term reduction in the effective stiffness of the PVB interlayer under sustained loading. (vi) EVA presents relatively smaller long-term values *σ_x_*, *τ_xy_* and *u_z_*, suggesting better long-term stiffness retention under the adopted material parameters. The long-term response of SGP generally lies between those of PVB and EVA. (vii) Although the final long-term response of SGP is intermediate, SGP reaches the long-term stable state much later than PVB and EVA. This indicates that SGP has a slower relaxation process. Therefore, before full relaxation is reached, SGP can retain a relatively higher effective shear stiffness at the same intermediate time.

As shown in Figure 5, (i) for different temperatures, *σ_x_* and *τ_xy_* exhibit nearly identical through-thickness distribution patterns. Their values at the upper and lower surfaces of the glass layers increase with increasing temperature. (ii) The temperature-induced variation of *σ_z_* is limited, indicating the weak sensitivity of the thickness-direction normal stress to interlayer relaxation. (iii) As the temperature rises, *τ_xz_* carried by the interlayer gradually decreases due to the relaxation of the interlayer, resulting in a redistribution of load transfer toward the glass layers.

### 3.4. Effects of the Number of Glass Layers and Aspect Ratio

**Figure 6:**
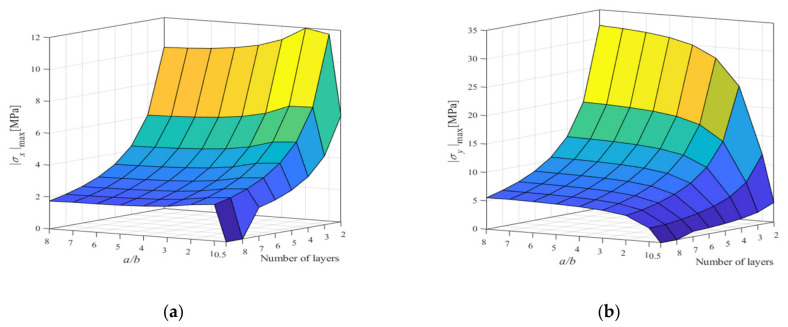

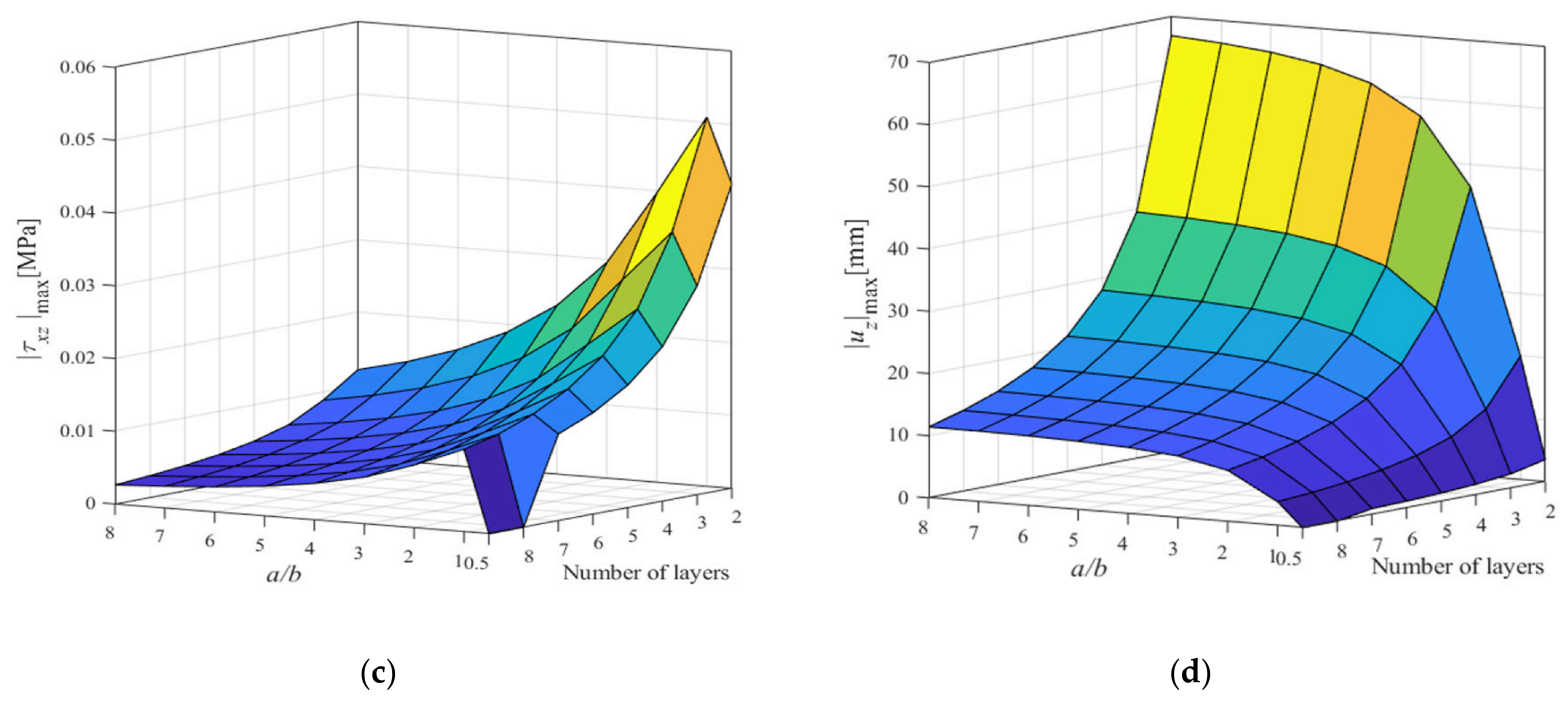
Effects of aspect ratio and number of glass layers on the maximum absolute values of *σ_x_*, *σ_y_*, *τ_xz_*, and *u_z_*. (**a**) $|\sigma_{x}{|}_{\max}$ varying with *a*/*b* and *p*; (**b**) $|\sigma_{y}{|}_{\max}$ varying with *a*/*b* and *p*; (**c**) $|\tau_{xz}{|}_{\max}$ varying with *a*/*b* and *p*; (**d**) $|u_{z}{|}_{\max}$ varying with *a*/*b* and *p*.

Effects of aspect ratio *a*/*b* and number of glass layers on the stresses and displacements of the LG plate are investigated. Figure 6 presents the variations of the maximum stress and maximum displacement responses $|\sigma_{x}{|}_{\max}$, $|\sigma_{y}{|}_{\max}$, $|\tau_{xz}{|}_{\max}$ and $|u_{z}{|}_{\max}$ with *a*/*b* and *p*, with *T* = 40 °C and Δ*h* = 0.76 mm.

It can be found from Figure 6 that (i) when *p* increases from 2 to 9, $|\sigma_{x}{|}_{\max}$, $|\sigma_{y}{|}_{\max}$, $|\tau_{xz}{|}_{\max}$ and $|u_{z}{|}_{\max}$ decrease rapidly at first and then decrease at a gradually reduced rate. This is because increasing p enhances the overall bending stiffness of the laminated structure. (ii) As *a*/b increases from 0.5 to 8, $|\sigma_{y}{|}_{\max}$ and $|u_{z}{|}_{\max}$ gradually increase and then tend to level off. In contrast, the $|\sigma_{x}{|}_{\max}$ and $|\tau_{xz}{|}_{\max}$ first increase rapidly and then decrease slowly, reaching their peak values within the small-to-moderate aspect-ratio range. (iii) The similar trends of $|\sigma_{x}{|}_{\max}$ and $|\tau_{xz}{|}_{\max}$ arise because both quantities are governed by bending in the *x*-direction and through-thickness shear transfer. In contrast, the consistent trends observed for $|\sigma_{y}{|}_{\max}$ and $|u_{z}{|}_{\max}$ arise because the plate response gradually shifts toward one-way, strip-like bending behavior as *a*/*b* increases. In this regime, the overall flexural deformation and the bending response in the *y*-direction become dominant.

### 3.5. Sensitivity Analysis of Viscoelastic Parameters

To evaluate the influence of uncertainties in the viscoelastic parameters, a sensitivity analysis is carried out for the PVB LG plate. The first Prony branch modulus *G*_1_*, the first relaxation time *θ*_1_, and the WLF constants *C*_1_ and *C*_2_ are selected as the perturbed parameters, while the other geometrical, material and loading parameters are kept unchanged. The perturbation levels *δ* are taken as −15% to +15%. For each case, the responses at *t* = 10^5^ s are recalculated. The relative variation of a response quantity is evaluated by Δ*R*/*R*_0_ = (*R*_δ_ − *R*_0_)/*R*_0_ × 100%, where *R*_δ_ and *R*_0_ denote the reference and corresponding perturbed values of *σ_x_*, *τ_xy_*, *τ_xz_* and *u_z_*.

As shown in Table 7, the perturbation of *G*_1_* causes only slight changes in the selected responses, while the perturbation of *θ*_1_ has almost no influence at the considered time. The WLF parameters *C*_1_ and *C*_2_ produce the largest variations, indicating their greater influence on the long-term viscoelastic response. Overall, the main conclusions remain stable within the considered perturbation range, although accurate WLF parameters are important for reliable long-term prediction.

## 4. Conclusions

An analytical solution is developed to evaluate the long-term bending response of multi-layer LG plates with temperature-dependent viscoelastic interlayers. Based on the numerical examples, the main conclusions can be drawn as follows.

1. The present solution shows rapid convergence when the Fourier truncation number is increased, and sufficient accuracy is achieved at *N* = 9. It also agrees well with the published long-term test and FE results, with average experimental errors below 4% and FE discrepancies within about 1.5%. Compared with the KL solution, the present model is more suitable for relatively thick plates, and it is about 1.13 × 10^3^ times faster than the FE simulation at *t* = 10^9^ s.
2. Temperature mainly affects the relaxation rate of the polymer interlayer rather than the final long-term response. A higher temperature accelerates the reduction of interlayer shear stiffness, causing the stress redistribution and deflection development to reach the long-term state earlier.
3. The interlayer material has a significant influence on the long-term composite action of LG plates. Compared with EVA and SGP, PVB leads to larger long-term deformation due to its lower stiffness retention, while stiffer interlayers provide stronger shear transfer and better bending resistance.
4. Increasing the number of glass layers improves the bending stiffness and reduces the stress and displacement responses. The aspect ratio also changes the bending behavior, making the plate response gradually approach one-way bending as the aspect ratio increases. The sensitivity analysis confirms that the main conclusions remain stable within the considered Prony and WLF parameter perturbation ranges.

## Figures and Tables

**Table 1:** WLF constants of different polymer interlayers at *T*_ref_ = 20 °C.

Polymer Interlayers	$C_{1}^{*}$	$C_{2}^{*}$[°C]
PVB	17.495	105.26
SGP	26.53	62.77
EVA	33.492	130.94

**Table 2:** Prony-series parameters of different polymer interlayers.

	PVB (*G*_0_* = 471 MPa)		SGP (*G*_0_* = 283.2 MPa)		EVA (*G*_0_* = 15.86 MPa)	
Prony-Series	G_j_*/*G*_0_*	$\theta_{j}$ [s]	G_j_*/*G*_0_*	$\theta_{j}$ [s]	G_j_*/*G*_0_*	$\theta_{j}$ [s]
*j* = 1	0.160	3.250 × 10^−11^	0.1219	13.32	0.1146	1.242 × 10^−10^
*j* = 2	0.07878	4.949 × 10^−9^	0.1095	185.7	0.06293	4.090 × 10^−9^
*j* = 3	0.2912	7.243 × 10^−8^	0.1726	2589	0.06260	1.346 × 10^−7^
*j* = 4	0.07116	9.864 × 10^−6^	0.1910	3.610 × 10^4^	0.05717	4.432 × 10^−6^
*j* = 5	0.2680	2.806 × 10^−3^	0.1787	5.032 × 10^5^	0.06026	1.459 × 10^−4^
*j* = 6	0.08959	0.1644	0.1262	7.016 × 10^6^	0.03688	4.803 × 10^−3^
*j* = 7	0.03018	2.255	0.06270	9.780 × 10^7^	0.04783	0.1581
*j* = 8	7.605 × 10^−3^	35.36	0.02053	1.363 × 10^9^	0.04167	5.204
*j* = 9	9.634 × 10^−4^	9.368 × 10^3^	6.534 × 10^−3^	1.901 × 10^10^	0.03171	1.713 × 10^2^
*j* = 10	4.059 × 10^−4^	6.414 × 10^5^	3.597 × 10^−3^	2.650 × 10^11^	0.03523	5.640 × 10^3^
*j* = 11	6.143 × 10^−4^	4.135 × 10^7^	2.647 × 10^−3^	3.694 × 10^12^	0.05195	1.857 × 10^5^
*j* = 12	/	/	1.498 × 10^−3^	5.150 × 10^13^	0.1285	6.112 × 10^6^
*j* = 13	/	/	1.372 × 10^−3^	7.179 × 10^14^	0.1881	2.012 × 10^8^

**Table 3:** Convergence of displacement and stress components with different Fourier truncation numbers.

*t* [s]	*N*	$\sigma_{x}$ [MPa]	$\tau_{xy}$ [MPa]	$\tau_{xz}$ [MPa]	$u_{z}$ [mm]
10^3^	1	12.36	−6.657	−0.07458	−14.11
	3	10.48	−7.451	−0.07959	−13.52
	5	10.92	−7.654	−0.08206	−13.58
	7	10.74	−7.734	−0.08238	−13.57
	9	10.83	−7.773	−0.08268	−13.57
10^6^	1	13.99	−7.530	−0.06151	−18.54
	3	11.81	−8.441	−0.06457	−17.79
	5	12.30	−8.661	−0.06578	−17.86
	7	12.10	−8.745	−0.06593	−17.85
	9	12.19	−8.785	−0.06606	−17.85
10^9^	1	18.98	−10.22	−0.02125	−32.22
	3	16.45	−11.27	−0.02184	−31.27
	5	16.97	−11.50	−0.02203	−31.34
	7	16.77	−11.59	−0.02205	−31.33
	9	16.87	−11.63	−0.02207	−31.33

**Table 4:** Comparisons of KL and FE results with the present results for different length-to-thickness ratios *a*/*H* when *t* = 10^9^ s.

*a*/*H*	Solutions	Present	KL	Error of KL	FE	Error of FE
5	$\sigma_{x}$ [MPa]	9.878	9.967	0.9030%	9.969	0.9172%
	$\tau_{xz}$ [MPa]	−3.160	−3.280	3.769%	−3.190	0.9335%
	$u_{z}$ [mm]	−0.01220	−0.0107	−12.30%	−0.01231	0.9180%
10	$\sigma_{x}$ [MPa]	9.734	9.694	−0.4130%	9.835	1.035%
	$\tau_{xz}$ [MPa]	−1.622	−1.643	1.015%	−1.638	1.005%
	$u_{z}$ [mm]	−0.04430	−0.04280	−3.386%	−0.04473	0.9752%
20	$\sigma_{x}$ [MPa]	9.707	9.688	−0.3901%	9.821	1.178%
	$\tau_{xz}$ [MPa]	−0.8187	−0.8198	0.1564%	−0.8280	1.136%
	$u_{z}$ [mm]	−0.1730	−0.1713	−1.081%	−0.1748	1.058%
100	$\sigma_{x}$ [MPa]	9.680	9.679	−0.01100%	9.819	1.436%
	$\tau_{xz}$ [MPa]	−0.1641	−0.1641	0	−0.1664	1.399%
	$u_{z}$ [mm]	−4.274	−4.272	−0.04210%	−4.337	1.467%

**Table 5:** Comparison of computational cost between the present solution and FE simulation for the LG plate with *a*/*H* = 100.

*t* [s]	Present CPU Time [s]	FE CPU Time [s]	Present Memory [MB]	FE Memory [MB]
10^3^	0.2203	295.3	80.17	690.1
10^6^	0.3378	400.1	87.35	768.6
10^9^	0.4570	517.6	91.78	843.5

**Table 6:** Comparison of stress and displacement responses between the interface conditions and the individual layer models for different interlayer thickness ratios at *t* = 10^6^ s and *t* = 10^9^ s.

Δ*h*/*h*_1_	*t* [s]	Solutions	Interface Conditions	Individual Layer	Error of Interface Conditions
0.152	10^6^	$\sigma_{x}$ [MPa]	31.248	27.36	−14.21%
		$\tau_{xz}$ [MPa]	−16.826	−14.73	−14.23%
		$u_{z}$ [mm]	−73.682	−61.22	−20.36%
	10^9^	$\sigma_{x}$ [MPa]	42.88	39.83	−7.658%
		$\tau_{xz}$ [MPa]	−23.09	−21.45	−7.646%
		$u_{z}$ [mm]	−137.3	−125.2	−9.665%
0.076	10^6^	$\sigma_{x}$ [MPa]	8.627	8.125	−6.179%
		$\tau_{xz}$ [MPa]	−4.645	−4.375	−6.171%
		$u_{z}$ [mm]	−11.44	−10.55	−8.436%
	10^9^	$\sigma_{x}$ [MPa]	11.71	11.44	−2.360%
		$\tau_{xz}$ [MPa]	−6.305	−6.161	−2.337%
		$u_{z}$ [mm]	−19.87	−19.32	−2.847%
0.038	10^6^	$\sigma_{x}$ [MPa]	2.426	2.369	−2.406%
		$\tau_{xz}$ [MPa]	−1.307	−1.275	−2.510%
		$u_{z}$ [mm]	−1.799	−1.744	−3.154%
	10^9^	$\sigma_{x}$ [MPa]	3.103	3.082	−0.6814%
		$\tau_{xz}$ [MPa]	−1.671	−1.660	−0.6627%
		$u_{z}$ [mm]	−2.724	−2.702	−0.8142%

**Table 7:** Relative variations of stress and displacement responses at *t* = 10^5^ s caused by perturbations of Prony-series and WLF parameters.

Perturbed Parameter	*δ*	$\Delta \sigma_{x}/\sigma_{x_{0}}$	$\Delta \tau_{xy}/\Delta \tau_{xy_{0}}$	$\Delta \tau_{xz}/\tau_{xz_{0}}$	$\Delta u_{z}/u_{z_{0}}$
*G*_1_* [MPa]	−15%	0.06242%	0.07374%	−0.3906%	0.1099%
	−10%	0.001388%	0.01203%	0.0000%	0.02446%
	−5%	0.05907%	0.05851%	−0.3906%	0.1047%
	5%	−0.004923%	−0.005485%	0.0000%	−0.007616%
	10%	0.05405%	0.05485%	−0.3906%	0.09734%
	15%	−0.008762%	−0.009142%	0.0000%	−0.01466%
$\theta_{1}$ [s]	−15%	0	0	0	0
	−10%	0	0	0	0
	−5%	0	0	0	0
	5%	0	0	0	0
	10%	0	0	0	0
	15%	0	0	0	0
$C_{1}^{*}$	−15%	7.335%	7.334%	−23.44%	13.04%
	−10%	5.356%	5.355%	−17.19%	9.525%
	−5%	2.907%	2.907%	−9.375%	5.171%
	5%	−3.265%	−3.266%	10.55%	−5.804%
	10%	−6.655%	−6.655%	21.09%	−11.83%
	15%	−9.822%	−9.822%	31.25%	−17.46%
$C_{2}^{*}$ [°C]	−15%	−9.480%	−9.480%	30.08%	−16.86%
	−10%	−6.100%	−6.100%	19.53%	−10.85%
	−5%	−2.851%	−2.851%	8.984%	−5.069%
	5%	2.379%	2.379%	−7.813%	4.231%
	10%	4.314%	4.313%	−13.67%	7.673%
	15%	5.870%	5.869%	−18.75%	10.44%

## Data Availability

The original contributions presented in this study are included in the article. Further inquiries can be directed to the corresponding author.

## Abbreviations and Notation

The following abbreviations are used in this manuscript:

LG	Laminated Glass
PVB	Polyvinyl Butyral
WLF	Williams–Landel–Ferry
FE	Finite Element
3D	Three-Dimensional
KL	Kirchhoff–Love
NB	No-Bonding
PB	Perfect-Bonding
SGP	SentryGlas Plus
EVA	Ethylene-Vinyl Acetate
*a*; *b*; *H*	length, width, and thickness of the plate, respectively.
*h*_i_; Δ*h*	thickness of the *i*-th layer and the interlayer, respectively.
*p*	number of glass layers in the plate.
*α*_m_; *β*_n_	wave numbers in the *x*- and *y*-directions, respectively.
$\sigma_{x}^{i};\;\sigma_{y}^{i};\;\sigma_{z}^{i}$	normal stress components of the *i*-th layer.
$\tau_{xy}^{i};\;\tau_{xz}^{i};\;\tau_{yz}^{i}$	shear stress components of the *i*-th layer.
$\gamma_{xy}^{i};\;\gamma_{xz}^{i};\;\gamma_{yz}^{i}$	shear strain components of the *i*-th layer.
$\varepsilon_{x}^{i};\;\varepsilon_{y}^{i};\;\varepsilon_{z}^{i}$	normal strain components of the *i*-th layer.
$u^{i};\;u_{xy}^{i};\;u_{xz}^{i};\;u_{yz}^{i}$	displacement components of the *i*-th layer in the *x*-, *y*-, and *z*-directions, respectively.
*E*; *G*; *ν*	elastic modulus, shear modulus, and Poisson’s ratio in the interlayer, respectively.
$G_{\infty }^{*};$ $G_{j}^{*}$ *G*_0_	long-term shear modulus, *j*-th Maxwell-branch modulus, and instantaneous shear modulus of the polymer interlayer, respectively.
*θ_j_*	relaxation time of the *j*-th Maxwell branch.
$\Phi^{i};\;L^{i}$	auxiliary elastic coefficients used in the state-space formulation.
$X^{i};\;X_{mn}^{i}$	state vector of the *i*-th layer and its Fourier coefficient, respectively.
$M^{i};\;D_{mn}^{i}$	differential operator matrix and state-space coefficient matrix of the *i*-th layer, respectively.
$K_{mn}$	transfer matrix of the polymer interlayer in the Laplace domain.
$S_{mn};\;S_{mn}^{**}$	global transfer matrix and its submatrices.
$T_{mn}^{i};\;T_{mn}^{i,k}$	transfer-related matrix and the matrix obtained by replacing the *k*-th column in Cramer’s rule, respectively.
$\omega_{mn}^{ik\lambda };\;\eta_{mn}^{i\lambda };\;s_{mn}^{i\beta };\;c_{\beta mn}^{ik}$	coefficients, characteristic roots, and corresponding residue coefficients used in the inverse Laplace-transform solution.
*Q_i_*	equivalent volumetric heat source of the *i*-th layer.
*I*_s_	incident solar radiation intensity.
*η*_i_	solar absorption ratio of the *i*-th layer.
*T*_in_; *T*_out_; *h*_in_; *h*_out_	outdoor and indoor air temperatures, and the corresponding convective heat transfer coefficients, respectively.
*χ*	aging variable of the interlayer.
*R*_0_; *R*_δ_	reference response and response after parameter perturbation, respectively.

## Appendix A

For comparison with the present three-dimensional elasticity solution, a KL plate solution is also developed. In this model, the two glass layers are described by the KL plate theory, while the polymer interlayer is idealized as a thin shear layer. The viscoelastic constitutive relation of the interlayer is the same as that given in Section 2.3 and is, therefore, not repeated here.

Consider a two-layer LG plate consisting of two glass layers and one polymer interlayer. The distance between the middle surfaces of the two glass layers is

(A1) $$d_{12}=\Delta h+\frac{h_{1}+h_{2}}{2},$$

For the *i*-th glass layer (*i* = 1, 2), the KL displacement field is expressed as

$$U_{i}(x,y,\zeta_{i},t)=u_{i}(x,y,t)-\zeta_{i}w_{,x}(x,y,t),$$

$$V_{i}(x,y,\zeta_{i},t)=v_{i}(x,y,t)-\zeta_{i}w_{,y}(x,y,t),$$

(A2) $$W_{i}(x,y,\zeta_{i},t)=w(x,y,t),$$

where *u_i_* and *v_i_* are the in-plane displacements of the middle surface of the *i*-th glass layer, *w* is the common transverse displacement of the two glass layers, and *ζ_i_* is the local thickness coordinate measured from the middle surface of the corresponding glass layer. The in-plane strain vector of the *i*-th glass layer is

(A3) $$\varepsilon^{\left(i\right)}=\varepsilon_{0}^{\left(i\right)}-\zeta_{i}\kappa ,$$

with

$$\varepsilon_{0}^{\left(i\right)}=\left[\begin{array}{c} u_{i,x}\\ v_{i,y}\\ u_{i,y}+v_{i,x} \end{array}\right],\;\kappa =\left[\begin{array}{c} w_{,xx}\\ w_{,yy}\\ 2w_{,xy} \end{array}\right].$$

The glass layers are assumed to be isotropic linear elastic materials under plane-stress conditions, namely

(A4) $$\sigma^{\left(i\right)}=Q^{\left(i\right)}\varepsilon^{\left(i\right)},$$

The membrane and bending stiffness matrices of the *i*-th glass layer are defined as

(A5) $$A^{\left(i\right)}=\int_{-h_{i}/2}^{h_{i}/2}Q^{\left(i\right)}d\zeta_{i}=h_{i}Q^{\left(i\right)},$$

(A6) $$D^{\left(i\right)}=\int_{-h_{i}/2}^{h_{i}/2}Q^{\left(i\right)}\zeta_{i}^{2}d\zeta_{i}=\frac{h_{i}^{3}}{12}Q^{\left(i\right)},$$

Therefore, the strain energy density of the two glass layers is written as

(A7) $$U_{g}=\frac{1}{2}\sum_{i=1}^{2}\left[{\left(\varepsilon_{0}^{\left(i\right)}\right)}^{T}A^{\left(i\right)}\varepsilon_{0}^{\left(i\right)}+\kappa^{T}D^{\left(i\right)}\kappa \right],$$

The interlayer is assumed to resist only transverse shear deformation. Its normal stress, normal strain and bending stiffness are neglected. The shear strain vector of the interlayer is defined from the relative in-plane displacement of the two glass layers and the plate rotations as

(A8) $$\gamma^{*}=\frac{1}{\Delta h}\left[\left[\begin{array}{c} u_{1}-u_{2}\\ v_{1}-v_{2} \end{array}\right]+d_{12}\left[\begin{array}{c} w_{,x}\\ w_{,y} \end{array}\right]\right],$$

For the corresponding elastic shear-layer problem, the interlayer shear energy density can be written as

(A9) $$U_{p}=\frac{G^{*}}{2\Delta h}\left[{\left(u_{1}-u_{2}+d_{12}w_{,x}\right)}^{2}+{\left(v_{1}-v_{2}+d_{12}w_{,y}\right)}^{2}\right],$$

For the viscoelastic interlayer considered in this study, *G** in Equation (A9) is replaced by the same hereditary shear operator defined in Section 2.3. In the Laplace-domain implementation, this is equivalent to introducing the viscoelastic shear coupling through the transformed relaxation modulus already used in the present formulation. The total potential energy of the KL reference plate is

(A10) $$\Pi =\int_{0}^{a}\int_{0}^{b}\left(U_{g}+U_{p}-q(x,y,t)w\right)dxdy,$$

The governing equations are obtained from the stationary condition

(A11) δΠ=0.

For the simply supported rectangular plate, these displacement components and the transverse load are expanded into double Fourier series satisfying the boundary conditions. Substitution into Equation (A11) gives a set of modal equations. The viscoelastic terms are then treated in the Laplace domain using the same interlayer constitutive relation as in Section 2.3, and the time-dependent response is obtained by inverse Laplace transformation.
